# Viral DNAemia and DNA Virus Seropositivity and Mortality in Pediatric Sepsis

**DOI:** 10.1001/jamanetworkopen.2024.0383

**Published:** 2024-02-26

**Authors:** Stephanie S. Cabler, Gregory A. Storch, Jason B. Weinberg, Andrew H. Walton, Karen Brengel-Pesce, Zachary Aldewereld, Russell K. Banks, Valerie Cheynet, Ron Reeder, Richard Holubkov, Robert A. Berg, David Wessel, Murray M. Pollack, Kathleen Meert, Mark Hall, Christopher Newth, John C. Lin, Tim Cornell, Rick E. Harrison, J. Michael Dean, Joseph A. Carcillo

**Affiliations:** 1 Department of Pediatrics, Washington University in St Louis, St Louis, Missouri; 2 Department of Pediatrics, University of Michigan, Ann Arbor; 3 bioMerieux, France; 4 Department of Pediatrics and Critical Care Medicine, University of Pittsburgh, Pittsburgh, Pennsylvania; 5 Department of Pediatrics, University of Utah, Salt Lake City; 6 Department of Anesthesiology, Pediatrics, University of Pennsylvania, Philadelphia; 7 Department of Pediatrics, George Washington University, Washington, DC; 8 Department of Pediatrics, Central Michigan University, Detroit; 9 Department of Pediatrics, The Ohio State University, Columbus; 10 Department of Anesthesiology, University of Southern California, Los Angeles; 11 Department of Pediatrics, University of California, Los Angeles

## Abstract

**Importance:**

Sepsis is a leading cause of pediatric mortality. Little attention has been paid to the association between viral DNA and mortality in children and adolescents with sepsis.

**Objective:**

To assess the association of the presence of viral DNA with sepsis-related mortality in a large multicenter study.

**Design, Setting, and Participants:**

This cohort study compares pediatric patients with and without plasma cytomegalovirus (CMV), Epstein-Barr virus (EBV), herpes simplex virus 1 (HSV-1), human herpesvirus 6 (HHV-6), parvovirus B19 (B19V), BK polyomavirus (BKPyV), human adenovirus (HAdV), and torque teno virus (TTV) DNAemia detected by quantitative real-time polymerase chain reaction or plasma IgG antibodies to CMV, EBV, HSV-1, or HHV-6. A total of 401 patients younger than 18 years with severe sepsis were enrolled from 9 pediatric intensive care units (PICUs) in the Eunice Kennedy Shriver National Institute of Child Health and Human Development Collaborative Pediatric Critical Care Research Network. Data were collected from 2015 to 2018. Samples were assayed from 2019 to 2022. Data were analyzed from 2022 to 2023.

**Main Outcomes and Measures:**

Death while in the PICU.

**Results:**

Among the 401 patients included in the analysis, the median age was 6 (IQR, 1-12) years, and 222 (55.4%) were male. One hundred fifty-four patients (38.4%) were previously healthy, 108 (26.9%) were immunocompromised, and 225 (56.1%) had documented infection(s) at enrollment. Forty-four patients (11.0%) died in the PICU. Viral DNAemia with at least 1 virus (excluding TTV) was detected in 191 patients (47.6%) overall, 63 of 108 patients (58.3%) who were immunocompromised, and 128 of 293 (43.7%) who were not immunocompromised at sepsis onset. After adjustment for age, Pediatric Risk of Mortality score, previously healthy status, and immunocompromised status at sepsis onset, CMV (adjusted odds ratio [AOR], 3.01 [95% CI, 1.36-6.45]; *P* = .007), HAdV (AOR, 3.50 [95% CI, 1.46-8.09]; *P* = .006), BKPyV (AOR. 3.02 [95% CI, 1.17-7.34]; *P* = .02), and HHV-6 (AOR, 2.62 [95% CI, 1.31-5.20]; *P* = .007) DNAemia were each associated with increased mortality. Two or more viruses were detected in 78 patients (19.5%), with mortality among 12 of 32 (37.5%) who were immunocompromised and 9 of 46 (19.6%) who were not immunocompromised at sepsis onset. Herpesvirus seropositivity was common (HSV-1, 82 of 246 [33.3%]; CMV, 107 of 254 [42.1%]; EBV, 152 of 251 [60.6%]; HHV-6, 253 if 257 [98.4%]). After additional adjustment for receipt of blood products in the PICU, EBV seropositivity was associated with increased mortality (AOR, 6.10 [95% CI, 1.00-118.61]; *P* = .049).

**Conclusions and Relevance:**

The findings of this cohort study suggest that DNAemia for CMV, HAdV, BKPyV, and HHV-6 and EBV seropositivity were independently associated with increased sepsis mortality. Further investigation of the underlying biology of these viral DNA infections in children with sepsis is warranted to determine whether they only reflect mortality risk or contribute to mortality.

## Key Points

**Question:**

Are viral DNAemia and DNA virus seropositivity associated with increased mortality in pediatric sepsis?

**Findings:**

In this multicenter cohort study of 401 children with severe sepsis, viral DNAemia was common, with at least 1 virus detected in 191 patients (48%). Cytomegalovirus, adenovirus, BK polyomavirus, and human herpesvirus 6 DNAemia and Epstein-Barr virus seropositivity were associated with increased mortality.

**Meaning:**

Further study is warranted to determine whether cytomegalovirus, adenovirus, BK polyomavirus, and human herpesvirus 6 DNAemia and Epstein-Barr virus seropositivity only reflect mortality risk or contribute to mortality in children with sepsis.

## Teaser

This cohort study assesses the association of mortality with DNA seropositivity and DNAemia among children and adolescents with severe sepsis in the United States.

## Introduction

Sepsis remains the leading cause of pediatric mortality worldwide. While sepsis recognition campaigns have improved early mortality,^1,2^ ongoing contributors to mortality are unclear. Most children with severe sepsis now die after initial resuscitative efforts due to development of progressive multiple organ failure in the pediatric intensive care unit (PICU).^1,3,4,5,6^ Detection of viral DNA in the bloodstream (viral DNAemia), presumed due to reactivation of latent infection, is an independent predictive factor associated with mortality in immunocompromised patients in the ICU.^7^ Viral DNAemia has also been detected in critically ill nonimmunocompromised patients, but the contribution to mortality in pediatric patients with sepsis remains unclear.^8,9,10,11,12,13,14^ While mechanisms triggering reactivation are not fully defined, conditions found in critical illness—including new infection, physiologic stress, immunosuppression, and trauma—have been implicated.^10,15,16^

Persistent and latent herpesvirus infections have been associated with human disease. Latent Epstein-Barr virus (EBV) infection is associated with autoimmune disease, including multiple sclerosis, posttransplant lymphoproliferative disorder, and post–COVID-19 conditions.^17,18,19,20^ Persistent and latent cytomegalovirus (CMV) infection has been associated with risk of cardiovascular disease, including ischemic heart disease, stroke, and cardiovascular death.^21,22^ Conversely, some evidence suggests that latent DNA virus infection increases resistance to other infections. For example, murine studies^23,24^ have shown that herpesvirus latency upregulates innate immunity and shields against certain bacterial infections, and that gammaherpesvirus latency protects against inflammatory responses in acute adenoviral disease, suggesting the association between host and latent virus may be symbiotic or even protective.

Previously, Davila et al^8^ studied viral DNAemia among patients in a single-center PICU. The investigators detected plasma DNAemia with multiple viruses and found associations with immunocompromised state, risk of secondary infection, and longer PICU stay and a trend toward increased mortality. Our present multicenter cohort investigation uses mortality as the primary outcome. We further measured selected herpesvirus antibody levels to elucidate a role for reactivation. We used biobanked samples collected from a parent study investigating pediatric sepsis phenotypes.^25^

## Methods

### Enrollment Criteria and Sepsis Definition

The parent study^25^ of the present cohort study included 401 patients from 9 PICUs in the Eunice Kennedy Shriver National Institutes of Child Health and Human Development Collaborative Pediatric Critical Care Research Network approved by the central institutional review board of the University of Utah. Enrollment criteria included severe sepsis diagnosis, presence of a central venous or arterial catheter, and age range from 44 weeks of gestation to younger than 18 years. Written informed consent was obtained from 1 or more parents or guardians for each child. Assent was obtained when the child was able. Parents self-identified child sex, race, ethnicity, and gender voluntarily. Race and ethnicity are considered important because they can affect outcome in children in relation to health disparity. Our research network was specifically conceived and designed by the Eunice Kennedy Shriver National Institutes of Child Health and Human Development to represent all racial and ethnic groups for equity in research participation. Severe sepsis was identified by suspected infection with abnormal heart rate, breathing, temperature, and/or white blood cell count with organ failure.^25^ Immunocompromised state at time of sepsis onset was based on underlying diseases or immunosuppressive treatments. Blood was collected from all enrolled patients twice per week to 28 days of admission or until death or PICU discharge. As a secondary analysis of a prior prospective study, our cohort study followed the Strengthening the Reporting of Observational Studies in Epidemiology (STROBE) reporting guideline.

### Viral DNA Testing

DNA was extracted from frozen plasma samples using an automated nucleic acid extractor (NucliSENS easyMag; bioMérieux). DNA was tested using quantitative real-time polymerase chain reaction (qPCR) assays (R-gene; bioMérieux) for the following viruses (limits of detection): CMV (290 copies/mL), EBV (182 copies/mL), herpes simplex virus 1 (HSV-1 [500 copies/mL]), human herpesvirus 6 (HHV-6 [200 copies/mL]), parvovirus B19 (B19V [200 copies/mL]), BK polyomavirus (BKPyV; [218 copies/mL]), human adenovirus (HAdV; [550 copies/mL]), and torque teno virus (TTV; [250 copies/mL]).^9^

### Serologic Testing

The earliest available plasma sample day from each patient’s PICU stay (sample 2 from the serial collection used for 275 patients, sample 1 used for 125 patients, sample 3 used for 1 patient) was simultaneously tested for both DNAemia of the 8 viruses and IgG antibodies to CMV, EBV (viral capsid antigen), HSV-1, and HHV-6. Testing was performed using multiplex flow immunoassays (BioPlex 2200 system; BioRad) except for HHV-6, which was tested using an indirect fluorescence assay (SCIMEDX Corporation). Results were expressed as positive, negative, or equivocal. When sufficient volume was available, tests with equivocal results were repeated. Patients younger than 18 months or who received intravenous immunoglobulin (IVIG) prior to testing were excluded from analysis of serologic results due to possible false-positive results from transplacental transfer of maternal antibody or from IVIG.

### Patient Classifications

Patients were defined as having viral DNAemia based on qPCR detection of 1 of the 8 viruses tested in 1 or more plasma samples. Because of its frequency and uncertain relationship to disease, TTV was not included in most analyses. Patients were defined as being seropositive based on detection of virus-specific IgG. For the 4 herpesviruses (CMV, EBV, HSV-1, and HHV-6), results of combined qPCR and serologic testing classified patients among 4 groups: no infection (IgG negative and DNAemia negative), acute infection (IgG negative and DNAemia positive), presumed reactivated infection (IgG positive and DNAemia positive), or latent infection without reactivation (IgG positive and DNAemia negative).

### Statistical Analysis

Data were collected from 2015 to 2018. Samples were assayed from 2019 to 2022. Data were analyzed from 2022 to 2023. Demographic characteristics, illness severity, PICU admission diagnoses, and specific medical history categories were summarized by immunocompromised state using counts (percentages) and medians (IQR) (Table). Proportions of patients with positive viral DNAemia and seropositivity were calculated among patients with testing results available. Mortality in the PICU was considered the primary outcome. Univariate analysis was used to identify variables associated with both mortality and presence of viral DNAemia or seropositivity for subsequent multivariate analysis. Adjusted odds ratios (AORs) and 95% CIs estimating the increase in odds of mortality associated with DNAemia were adjusted for age, Pediatric Risk of Mortality (PRISM) score at ICU admission, whether or not the patient was deemed healthy prior to hospitalization, and immunocompromised status. The AOR for increased mortality associated with seropositivity was additionally adjusted for receipt of blood products in the PICU prior to sampling for antibody measurement. All logistic regression analyses were performed in SAS, version 9.4 (SAS Institute). Select figures were generated using R, version 4.2.1, and package ggplot (R Project for Statistical Computing). *P* values were based on a 2-sided alternative and considered significant if less than .05. No adjustments for multiple comparisons were made.

**Table.** Study Population

Characteristic	Patient group^a^		
	All (N = 401)	Nonimmunocompromised (n = 293)	Immunocompromised (n = 108)^b^
Age, median (IQR), y	6 (1-12)	5 (1-12)	8 (4-13)
Sex			
Female	179 (44.6)	134 (45.7)	45 (41.7)
Male	222 (55.4)	159 (54.3)	63 (58.3)
Race			
Black or African American	83 (20.7)	61 (20.8)	22 (20.4)
White	270 (67.3)	195 (66.6)	75 (69.4)
Multiracial	3 (0.7)	3 (1.0)	0
Other^c^	19 (4.7)	13 (4.4)	6 (5.6)
Unknown or not reported	26 (6.5)	21 (7.2)	5 (4.6)
Ethnicity			
Hispanic or Latino	66 (16.5)	47 (16.0)	19 (17.6)
Non-Hispanic or non-Latino	321 (80.0)	234 (79.9)	87 (80.6)
Unknown or not reported	14 (3.5)	12 (4.1)	2 (1.9)
PRISM score, median (IQR)^d^	8 (3-14)	7 (3-13)	10 (6-16)
Infection at eligibility^e^			
Documented infection	225 (56.1)	166 (56.7)	59 (54.6)
Bacterial	141 (35.2)	102 (34.8)	39 (36.1)
Fungal	4 (0.9)	1 (0.3)	3 (2.8)
Protozoal	1 (0.2)	1 (0.3)	0
Viral^f^	112 (27.9)	87 (29.7)	25 (23.1)
PICU admission diagnosis			
Cardiac arrest	12 (3.0)	11 (3.8)	1 (0.9)
Cardiac disease	18 (4.5)	15 (5.1)	3 (2.8)
Liver failure	5 (1.2)	5 (1.7)	0
Kidney failure	9 (2.2)	4 (1.4)	5 (4.6)
Respiratory failure	166 (41.4)	136 (46.4)	30 (27.8)
Seizure	14 (3.5)	8 (2.7)	6 (5.6)
Sepsis	124 (30.9)	75 (25.6)	49 (45.4)
Other neurologic disease	15 (3.7)	13 (4.4)	2 (1.9)
Other	38 (9.5)	26 (8.9)	12 (11.1)
Medical history			
Previously healthy^g^	154 (38.4)	150 (51.2)	4 (3.7)
Hematologic malignant neoplasm	35 (8.7)	0	35 (32.4)
Solid tumor	16 (4.0)	1 (0.3)	15 (13.9)
Hematopoietic stem cell transplant	23 (5.7)	0	23 (21.3)
Solid organ transplant	10 (2.5)	0	10 (9.3)
Genetic disorder or chromosomal abnormality	63 (15.7)	47 (16.0)	16 (14.8)
Metabolic or mitochondrial disease	18 (4.5)	13 (4.4)	5 (4.6)

Abbreviations: PICU, pediatric intensive care unit; PRISM, Pediatric Risk of Mortality.

^a^ Unless otherwise indicated, data are expressed as No. (%) of patients. Percentages have been rounded and may not total 100.

^b^ Conditions included hematologic malignant neoplasm (n = 35), solid tumor (n = 16), hematopoietic stem cell transplant (n = 23), solid organ transplant (n = 10), chronic corticosteroid use (n = 59), congenital immunodeficiency (n = 10), rheumatologic disease (n = 7), functional or anatomic asplenia (n = 3), or other (n = 19). Patients could have more than 1 diagnosis; hence the total is greater than the total number of immunocompromised patients.

^c^ Includes self-identified American Indian or Alaska Native, Asian, and Native Hawaiian or Other Pacific Islander according to the National Institutes of Health demographic sheet provided to parents at the time of consent.

^d^ The worst physiologic values 2 hours prior to intensive care unit (ICU) admission through 4 hours post ICU admission were used to calculate PRISM. Scores range from 1 to 26, with higher scores indicating higher mortality risk.

^e^ Patients may have had multiple documented infections at eligibility; counts are not mutually exclusive.

^f^ Viral infection(s) at eligibility was determined by respective research coordinator medical record review and does not include results of investigative viral testing subsequently performed for this study.

^g^ Four patients were listed as both previously healthy and immunocompromised by the site research coordinator. Since no further information was available, patients were reported and analyzed as designated.

## Results

### Study Population

The study population of 401 patients is described in the Table. Of these, 179 patients (44.6%) were female and 222 (55.4%) were male; the median age was 6 (IQR, 1-12) years. In terms of race and ethnicity, 83 patients (20.7%) were Black, 66 (16.5%) were Hispanic, 270 (67.3%) were White, 3 (0.7%) were multiracial, 19 (4.7%) were of other race or ethnicity (including self-identified American Indian or Alaska Native, Asian, and Native Hawaiian or other Pacific Islander), and 26 (6.5%) were of unknown race or ethnicity. Only 154 patients (38.4%) were previously healthy; 108 (26.9%) were immunocompromised, and 225 (56.1%) had documented infection(s) at enrollment. The most common underlying diseases or conditions were immunocompromised state, genetic disorder or chromosomal abnormality, and metabolic or mitochondrial disease. Investigative viral testing performed for this study was not used for defining baseline disease state.

### Viral DNAemia

**Figure 1.**
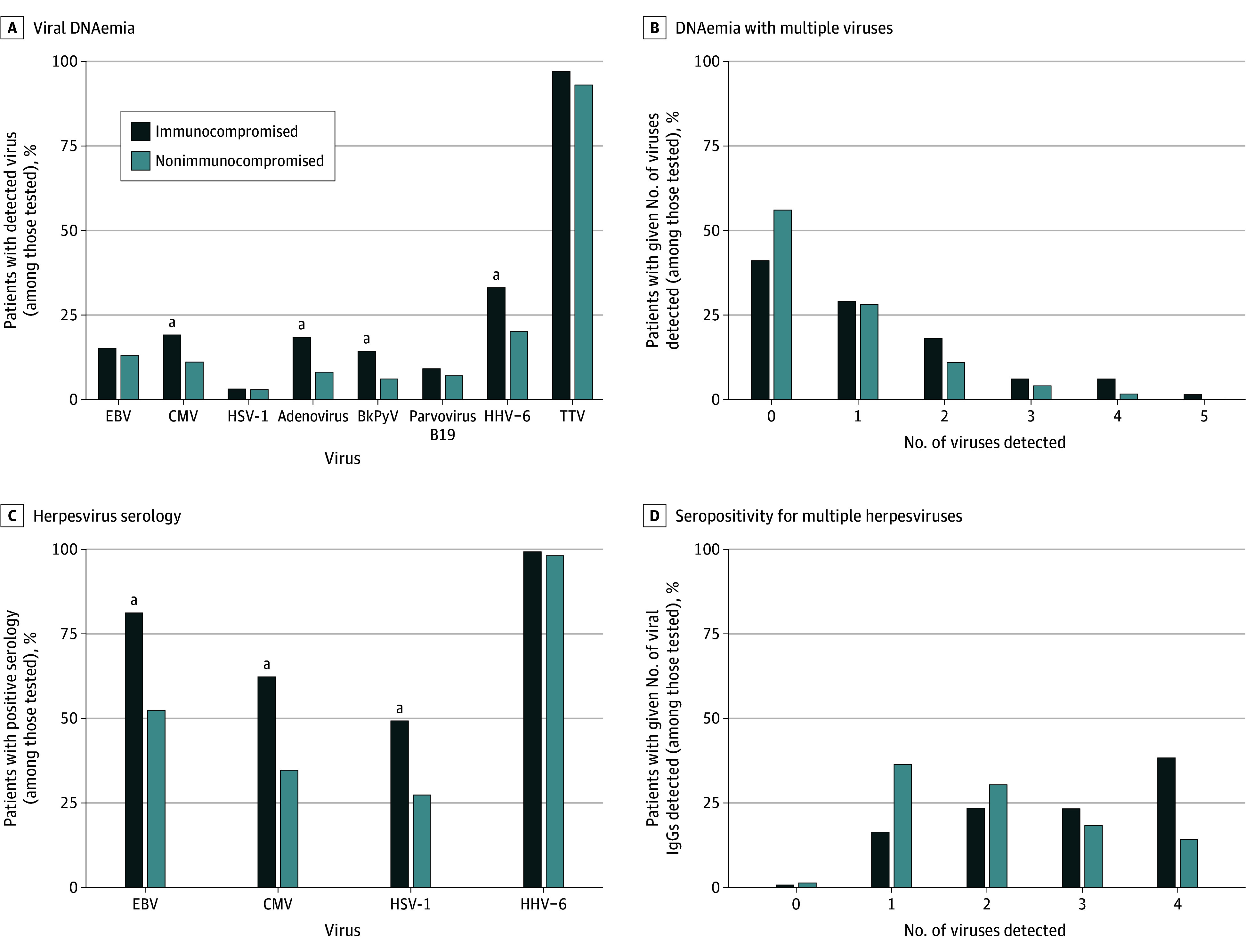
Viral DNAemia and Seropositivity by Immunocompromised Status A detailed breakdown of patient characteristics and all statistical comparisons can be found in eTable 1 in Supplement 1. The analysis of the number of viruses detected via polymerase chain reaction analysis excludes torque teno virus (TTV). Patients younger than 18 months or those who had intravenous immunoglobulin administration on the same day or before serology sample collection are excluded from serology analyses, leaving a sample size of 264. BkPyV indicates BK polyomavirus; CMV, cytomegalovirus; EBV, Epstein-Barr virus; HHV-6, human herpesvirus 6; and HSV-1, herpes simplex virus 1. ^a^*P* ≤ .01.

Figure 1A shows the occurrence of viral DNAemia and Figure 1B shows the number of viral DNAemias detected in patients who were or were not immunocompromised at the time of sepsis onset. Excluding TTV, 191 (47.6%) of all patients had viral DNAemia detected (63 of 108 [58.3%] who were and 128 of 293 [43.7%] who were not immunocompromised at sepsis onset), including 113 (28.2%) with 1 virus detected and 78 (19.5%) with more than 1 virus detected. The most frequently detected viral DNA was TTV, present in 368 of 391 patients (94.1%), followed by HHV-6 in 93 of 391 (23.8%), EBV in 53 of 385 (13.8%), CMV in 52 of 391 (13.3%), HAdV in 40 of 381 (10.5%), BKPyV in 32 of 385 (8.3%), B19V in 28 of 381 (7.3%), and HSV-1 in 11 of 385 patients (2.9%). Human adenovirus, BKPyV, and HHV-6 were each more likely to be detected in immunocompromised patients. Associations between viral DNAemia and demographic characteristics, illness severity, presence of infection at study entry, and ICU admission diagnoses are shown in eTable 1 in Supplement 1.

### Viral Serology

After excluding 108 patients who were younger than 18 months and 29 who received IVIG prior to their sample being obtained, 264 patients remained for analysis of herpesvirus serologic testing. Viral seropositivity was common, ranging from 82 of 246 (33.3%) for HSV-1 to 107 of 254 (42.1%) for CMV, 152 of 251 (60.6%) for EBV, and 253 of 257 (98.4%) for HHV-6. Percentage positivity for each virus by immunocompromised status are shown in Figure 1C, and percentage of viral IgGs detected are shown in Figure 1D. Seventy-seven patients were seropositive for 1 virus, 72 for 2, 51 for 3, and 55 for all 4 herpesviruses. Patients who were immunocompromised at sepsis onset were more likely than nonimmunocompromised patients to be seropositive for EBV, CMV, and HSV-1. Associations between herpesvirus serologic status and demographic characteristics, illness severity, presence of infection at study entry, and PICU admission diagnoses are shown in eTable 2 in Supplement 1.

### Combined qPCR and Serology for Herpesviruses

A detailed breakdown of results of qPCR and serologic testing is provided in eTable 3 in Supplement 1. Presence of viral DNAemia was characterized as presumed reactivation (seropositive with viral DNAemia) rather than primary infection (seronegative with viral DNAemia) in 39 of 43 patients (90.7%) with EBV, 19 of 30 (63.3%) with CMV, 8 of 8 (100%) with HSV-1, and 61 of 61 (100%) with HHV-6. The percentage of seropositive patients who had evidence of presumed reactivation was 39 of 152 (25.7%) for EBV, 19 of 107 (17.8%) for CMV, 8 of 82 (9.8%) for HSV-1, and 61 of 253 (24.1%) for HHV-6.

### Mortality

Of the 401 patients, 44 (11.0%) died while in the PICU. eTable 4 in Supplement 1 shows differences in univariate analysis between those who did or did not die. Patients who died were older, although the difference was not statistically significant (median age, 7.9 [IQR, 1.2-14.5] vs 5.4 [IQR, 1.4-12.0] years; *P* = .08), and had higher baseline PRISM scores^26,27^ (median, 13.0 [IQR, 8.5-18.5] vs 7.0 [IQR, 3.0-13.0]; *P* = .001). Patients who were immunocompromised at sepsis onset were more likely to die than those who were not immunocompromised at sepsis onset (21 of 108 [19.4%] vs 23 of 293 [7.8%]; *P* = .002).

### Viral DNAemia and Mortality

**Figure 2.**
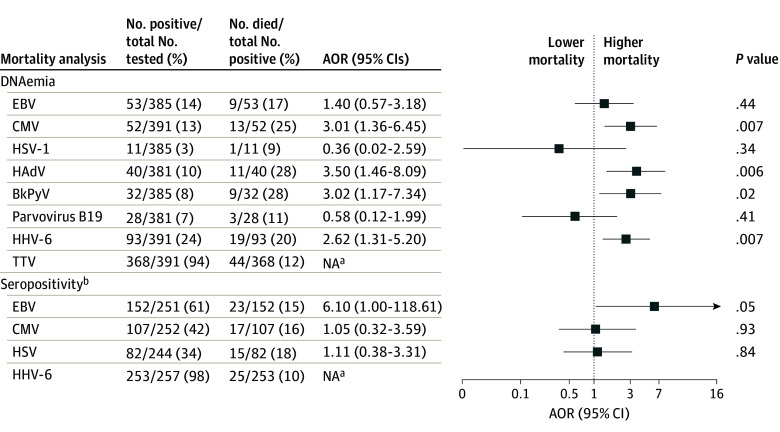
Forest Plot Showing Adjusted Odds Ratios (AORs) for Mortality Associations With Viral DNAemia and Seropositivity Odds ratios were adjusted for age, Pediatric Risk of Mortality (PRISM) score, previously healthy status, and immunocompromised status. Forest plots are presented on log scale due to large confidence intervals for some estimates. BkPyV indicates BK polyomavirus; CMV, cytomegalovirus; EBV, Epstein-Barr virus; HHV-6, human herpesvirus 6; HSV-1, herpes simplex virus 1; NA, not applicable; and TTV, torque teno virus. ^a^Missing AORs indicate that an insufficient number of patients were available to support the logistic regression model. ^b^Excludes patients younger than 18 months and/or those who received intravenous immunoglobulin prior to sample collection. In addition to age, PRISM score, having been previously healthy, and immunocompromised status, receipt of blood products prior to serology sample collection is also included as a covariate.

**Figure 3.**
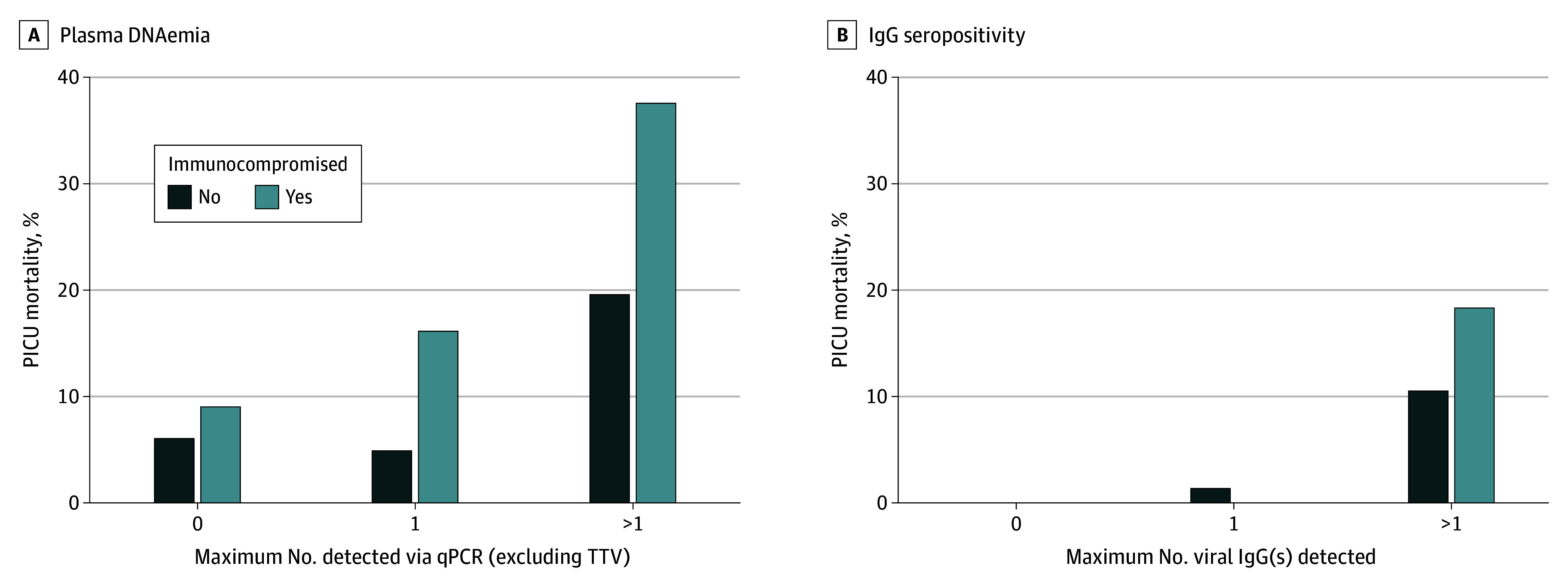
Pediatric Intensive Care Unit (PICU) Mortality by Number of Viruses Detected by Plasma Quantitative Real-Time Polymerase Chain Reaction (qPCR) Analysis and Herpesvirus Serology TTV indicates torque teno virus.

Adjusted mortality according to the presence of viral DNAemia is shown in Figure 2. After adjusting for age, PRISM score, presence of underlying diseases, and immunocompromised status at sepsis onset, increased mortality was observed in patients with DNAemia due to CMV (AOR, 3.01 [95% CI, 1.36-6.45]; *P* = .007), HAdV (AOR, 3.50 [95% CI, 1.46-8.09]; *P* = .006), BKPyV (AOR, 3.02 [95% CI, 1.17-7.34]; *P* = .02), and HHV-6 (AOR, 2.62 [95% CI, 1.31-5.20]; *P* = .007). Mortality in patients with viral DNAemia with more than 1 virus included 12 of 32 patients (37.5%) who were immunocompromised and 9 of 46 patients (19.6%) who were not immunocompromised at sepsis onset (Figure 3A). Detection of DNAemia with 3 or more viruses increased risk of death with morality in 10 of 28 patients (35.7%). Higher viral loads for each individual virus, including TTV, were not associated with increased mortality.

### Viral Serology and Mortality

As shown in Figure 2, after adjusting for age, PRISM score, presence of underlying diseases, immunocompromised status at sepsis onset, and receipt of blood products prior to serology sampling in the PICU, only patients seropositive for EBV but not those who were seropositive for the other 3 herpesviruses had increased mortality (AOR for EBV, 6.10 [95% CI, 1.00-118.60]; *P* = .049). Only 2 of 99 EBV-seronegative children (2.0%) died in the ICU compared with 23 of 152 (15.1%) who were EBV seropositive. Mortality was higher in patients with more than 1 herpesvirus IgG detected (12 of 65 [18.5%] in patients who were and 12 of 113 [10.6%] in patients who were not immunocompromised at sepsis onset) (Figure 3B).

### Combined qPCR and Serology Association With Mortality

**Figure 4.**
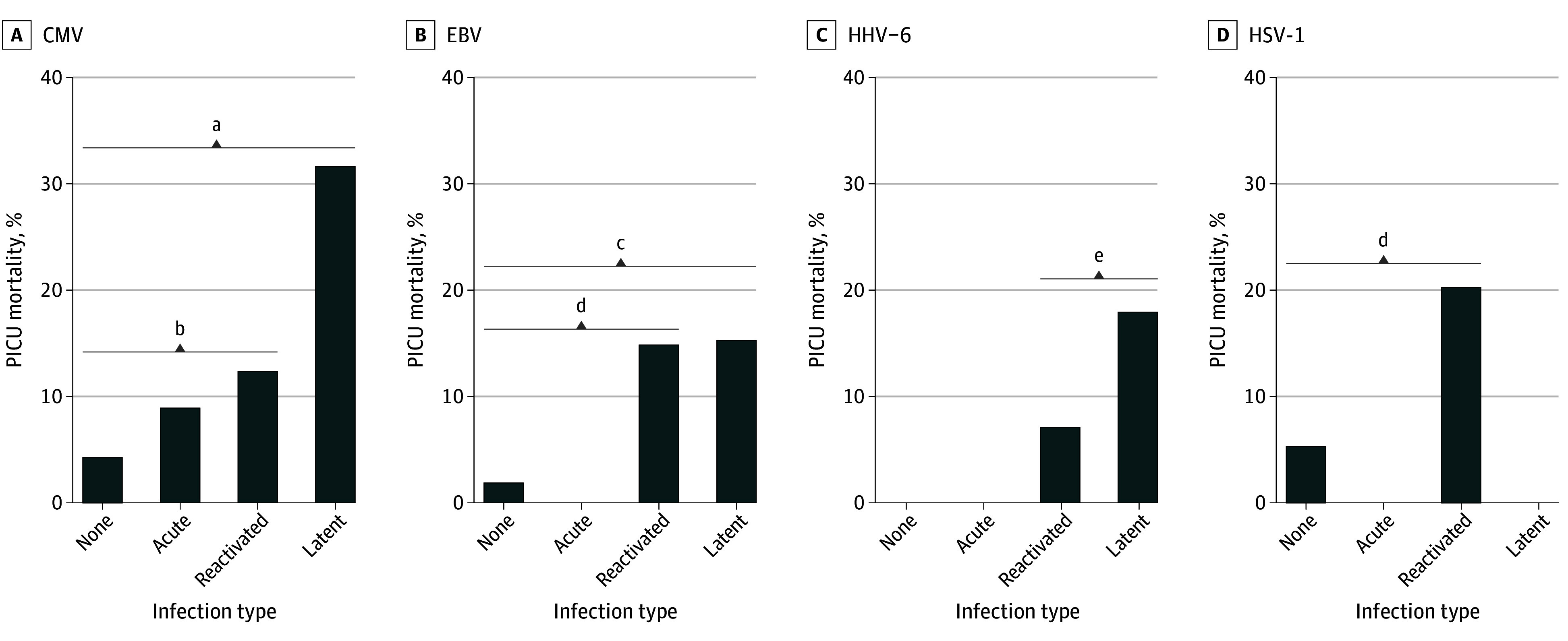
Association Among Plasma Quantitative Real-Time Polymerase Chain Reaction Analysis, Viral Seropositivity, and Mortality All pairwise comparisons were performed and can be found in eTable 5 in Supplement 1. *P* values were not adjusted for multiple comparisons. CMV indicates cytomegalovirus; EBV, Epstein-Barr virus; HHV-6, human herpesvirus 6; and HSV-1, herpes simplex virus 1. No infection indicates IgG negative and DNAemia negative; acute infection, IgG negative and DNAemia positive; reactivated infection, IgG positive and DNAemia positive; and latent infection, IgG positive and DNAemia negative. ^a^*P* = .04, no infection vs latent infection. ^b^*P* < .001, no infection vs reactivated infection. ^c^*P* = .008, no infection vs reactivated infection. ^d^*P* = .001, no infection vs latent infection. ^e^*P* = .02, latent infection vs reactivated infection.

Mortality among all patient classifications described in the Methods section is displayed in Figure 4, with a more detailed breakdown in eTables 5 to 8 in Supplement 1. For each virus, mortality was higher in seropositive patients compared with those who were seronegative, with low mortality in patients uninfected with specific viruses (EBV, 2 of 95 [2.1%]; CMV, 6 of 136 [4.4%]; HHV-6, 0 of 4; HSV-1, 9 of 164 [5.5%]). In patients with CMV, those with reactivation (1 of 11 [9.1%]) or latent infection (11 of 88 [12.5%]) were more likely to die than those without infection. Patients with latent EBV or HSV-1 infection were more likely to die than those without infection for the corresponding virus (for EBV, 17 of 113 [15.0%] vs 2 of 95 [2.1%]; for HSV-1, 15 of 74 [20.3%] vs 9 of 164 [5.5%]). Patients who were seropositive for CMV or HHV-6 each had higher mortality if they also had DNAemia with the corresponding virus. In contrast, EBV-seropositive patients had the same mortality (17 of 113 [15.0%] and 6 of 39 [15.4%]), regardless of the presence of EBV DNAemia. eTables 9 to 12 in Supplement 1 show ferritin and C-reactive protein levels.

## Discussion

Previously published single-center studies have reported frequent detection of viral DNAemia in adult^10,12,13,28,29,30^ and pediatric^8,11^ patients with sepsis that is correlated with immunocompromised state and severity of illness. Our present large multicenter cohort study validates and extends those findings. We report herein that viral DNAemia was common in pediatric patients admitted to the PICU with severe sepsis, including those who were and were not immunocompromised at the time of sepsis onset. In both groups, mortality increased with increasing number of viral DNAs detected in plasma using qPCR. In addition, CMV, HAdV, HHV-6, and BKPyV plasma DNAemia were each independently associated with increased mortality.

Analysis of herpesvirus serologic status in combination with measurements of viral DNAemia allowed separation into 4 distinct groups: uninfected, acute infection, latent infection, and presumed reactivation. DNAemia with any of the 4 herpesviruses was much more common in those who were seropositive for that virus compared with those who were seronegative, consistent with reactivation, and suggesting that primary infection in this setting was uncommon. We also found that seropositivity for EBV but not for any of the other 3 herpesviruses was associated with increased mortality in multivariate analysis. Only 2.0% of patients who were seronegative for EBV died compared with 15.1% of those who were seropositive for EBV. Possible explanations may be that EBV seropositivity is a biomarker for more severe illness that may not have been fully accounted for by the multivariate analysis or, alternatively, that being latently infected by EBV renders an individual more vulnerable to mortality from sepsis.

In addition to expanding the number of study participants compared with previous pediatric studies, this study also expanded the number of DNA viruses tested, adding BKPyV and B19V to those tested by Davila et al.^8^ As expected, not all DNA viruses behaved similarly. While CMV, HAdV, HHV-6, and BKPyV DNAemia conferred a significant risk of mortality, plasma DNAemia with HSV-1 and B19V were detected less often than the other viruses and were not associated with mortality. Our findings align with reports that only 2% to 10% of children are exposed to B19V before 5 years of age and that clinically significant reactivation is rare.^31,32,33,34^

Torque teno virus is commonly detected in patients in the ICU,^9^ and studies have suggested a direct correlation of TTV level with degree of T-cell dysfunction in patients undergoing hematopoietic cell transplant^35^ and solid organ transplant.^36^ Previous studies have not examined the association between TTV and mortality; our present study did not find higher levels of TTV associated with mortality.

The origin of viral DNAemia is currently not fully understood. We presumed that detection of viral DNAemia was related to reactivation from latency with new viral replication and production of infectious virus, possibly resulting from sepsis-induced immune suppression. However, detection of viral DNA could also result from release of nonviable viral DNA from latently infected cells that are damaged during sepsis. Assays that detect viable virus or other indicators of viral replication, such as viral messenger RNAs or viral proteins associated with active infection, could help clarify this issue.

Another important outstanding question is whether viral reactivation has adverse effects on patients with sepsis and possibly contributes to adverse outcomes, including death. A direct way to assess contributions of viral reactivation to outcomes of sepsis is to evaluate effects of antiviral treatment. Limaye et al^37^ performed a prospective double-blind, placebo-controlled randomized trial of ganciclovir in adult patients undergoing critical care who were at risk for CMV reactivation by virtue of being CMV seropositive and had either sepsis or trauma with respiratory failure. Antiviral therapy did not reduce plasma interleukin 6 levels but was associated with a reduction in CMV DNAemia and an increase in ventilator-free days. There was no impact on mortality. Papazian et al^38^ performed a similar double-blind, placebo-controlled randomized trial of 76 adults with CMV reactivation requiring mechanical ventilation and demonstrated no benefit in ventilator-free days in the group treated with preemptive ganciclovir. These results suggest that reactivated viral infection could be a risk biomarker for severity of sepsis rather than a direct contributor.

### Strengths and Limitations

Strengths of our study include a large multicenter population of children with sepsis and the use of serologic testing to evaluate whether herpesvirus DNAemia was a result of acute infection or reactivation from latency and to define a possible role for latent infection in sepsis pathogenesis. Our study also has important limitations. The number of children who died was small compared with deaths in studies of adults, leading to relatively wide 95% CIs in multivariable analyses. We lacked access to samples to assess frequency of viral DNAemia and seropositivity in non–critically ill children. Nevertheless, the herpesvirus seropositivity rates we found in our assayable pediatric sepsis samples were similar to those previously reported in age-matched healthy pediatric patients,^39,40,41,42,43,44,45,46^ implying that seropositive status may not be increased in children with sepsis as much as it is increased in children with higher proclivity to die of sepsis. We were unable to assess whether DNA virus reactivation occurred in specific locations such as the respiratory tract, which might not be detected in plasma.^16,29,47^ This could have led us to an underestimation of the frequency of DNA virus reactivation. We also lacked access to data regarding blood products received before the PICU stay. Although blood-bank filtering techniques all but eliminate passive transfer of viral DNAemia during transfusion, IgG antibody transfer can occur. Larger studies designed to account for blood product receipt prior to PICU admission and sepsis onset are needed to validate our findings.

## Conclusions

Our cohort study found that viral DNAemia was both common in pediatric patients with severe sepsis and independently associated with mortality. Cytomegalovirus, HAdV, BKPyV, and HHV-6 DNAemia were each significantly associated with mortality. Detection of viral DNAemia with multiple viruses during PICU admission increased the risk of mortality in patients who were or were not immunocompromised at the time of sepsis onset. Seropositivity for EBV but not for the other 3 herpesviruses studied was also associated with increased mortality and was not altered by whether or not the patient had EBV DNAemia. Our study does not address any causal nature of these associations, but our findings do identify a group of children at risk for sepsis mortality. While unrecognized confounding may explain the associations, active or latent infection with DNA viruses may reflect severity of illness or exert an adverse effect on the ability of children to respond to sepsis. Further investigation of underlying biology of these viral DNA infections in children with sepsis is warranted.

## Notes

Supplement 1. **eTable 1.** Patient Characteristics at ICU Admission by Virus Detection **eTable 2.** Patient Characteristics at ICU Admission by Results of Viral Serologic Testing **eTable 3.** Comparisons of Herpesvirus PCR and Serology Status **eTable 4.** Univariable Analysis of Admission Demographic and Clinical Factors Associated With Mortality **eTable 5.** Comparisons of EBV PCR and Serology Status and Mortality **eTable 6.** Comparisons of CMV PCR and Serology Status and Mortality **eTable 7.** Comparisons of HSV-1 PCR and Serology Status and Mortality **eTable 8.** Comparisons of HHV-6 PCR and Serology Status and Mortality **eTable 9.** Comparison of Ferritin, CRP, and PRISM Across EBV Virus Positivity Cohorts in Vital Status Categories **eTable 10.** Comparison of Ferritin, CRP, and PRISM Across CMV Virus Positivity Cohorts in Vital Status Categories **eTable 11.** Comparison of Ferritin, CRP, and PRISM Across HSV-1 Virus Positivity Cohorts in Vital Status Categories **eTable 12.** Comparison of Ferritin, CRP, and PRISM Across HHV-6 Virus Positivity Cohorts in Vital Status Categories

Supplement 2.
Data Sharing Statement
