# Errors in Figure

**DOI:** 10.1001/jamanetworkopen.2024.9203

**Published:** 2024-03-27

In the Original Investigation titled “Viral DNAemia and DNA Virus Seropositivity and Mortality in Pediatric Sepsis,”^1^ published February 26, 2024, the x-axis labels “Reactivated” and “Latent” were inadvertently transposed in Figure 4A-D, and the comparisons in footnotes a through e were incorrect. This article has been corrected.
