# Supplementary material for: Viral DNAemia and DNA Virus Seropositivity and Mortality in Pediatric Sepsis
Source: JAMA Netw Open. 2024 Feb 26;7(2):e240383. doi: 10.1001/jamanetworkopen.2024.0383 (PMC10897747; doi:10.1001/jamanetworkopen.2024.0383)
Supplement: Supplement 1. — eTable 1. Patient Characteristics at ICU Admission by Virus Detection eTable 2. Patient Characteristics at ICU Admission by Results of Viral Serologic Testing eTable 3. Comparisons of Herpesvirus PCR and Serology Status eTable 4. Univariable Analysis of Admission Demographic and Clinical Factors Associated With Mortality eTable 5. Comparisons of EBV PCR and Serology Status and Mortality eTable 6. Comparisons of CMV PCR and Serology Status and Mortality eTable 7. Comparisons of HSV-1 PCR and Serology Status and Mortality eTable 8. Comparisons of HHV-6 PCR and Serology Status and Mortality eTable 9. Comparison of Ferritin, CRP, and PRISM Across EBV Virus Positivity Cohorts in Vital Status Categories eTable 10. Comparison of Ferritin, CRP, and PRISM Across CMV Virus Positivity Cohorts in Vital Status Categories eTable 11. Comparison of Ferritin, CRP, and PRISM Across HSV-1 Virus Positivity Cohorts in Vital Status Categories eTable 12. Comparison of Ferritin, CRP, and PRISM Across HHV-6 Virus Positivity Cohorts in Vital Status Categories [file jamanetwopen-e240383-s001.pdf]

Supplementary Online Content

Cabler SS, Storch GA, Weinberg JB, et al. Viral DNAemia and DNA virus seropositivity and mortality in pediatric sepsis. *JAMA Netw Open*. 2024;7(2):e240383. doi:10.1001/jamanetworkopen.2024.0383

eTable 1. Patient Characteristics at ICU Admission by Virus Detection
eTable 2. Patient Characteristics at ICU Admission by Results of Viral Serologic Testing
eTable 3. Comparisons of Herpesvirus PCR and Serology Status
eTable 4. Univariable Analysis of Admission Demographic and Clinical Factors Associated With Mortality
eTable 5. Comparisons of EBV PCR and Serology Status and Mortality
eTable 6. Comparisons of CMV PCR and Serology Status and Mortality
eTable 7. Comparisons of HSV-1 PCR and Serology Status and Mortality
eTable 8. Comparisons of HHV-6 PCR and Serology Status and Mortality
eTable 9. Comparison of Ferritin, CRP, and PRISM Across EBV Virus Positivity Cohorts in Vital Status Categories
eTable 10. Comparison of Ferritin, CRP, and PRISM Across CMV Virus Positivity Cohorts in Vital Status Categories
eTable 11. Comparison of Ferritin, CRP, and PRISM Across HSV-1 Virus Positivity Cohorts in Vital Status Categories
eTable 12. Comparison of Ferritin, CRP, and PRISM Across HHV-6 Virus Positivity Cohorts in Vital Status Categories

This supplementary material has been provided by the authors to give readers additional information about their work.

© 2024 Cabler SS et al. *JAMA Network Open*.

eTable 1. Patient Characteristics at ICU Admission by Virus Detection

	EBV+ (N = 53)	CMV+ (N = 52)	HSV-1+ (N = 11)	Adeno+ (N = 40)	BKPyV+ (N = 32)	Parvo+ (N = 28)	HHV-6+ (N = 93)	TTV+ (N = 368)
**DEMOGRAPHICS**								
Age (years)	10 [3, 13]^γ	6 [1, 11]	12 [10, 15]^γ	6 [2, 12]	8 [4, 12]	12 [5, 15]^γ	6 [1, 12]	6 [1, 12]
Sex: Male	29 (55%)	28 (54%)	6 (55%)	24 (60%)	16 (50%)	18 (64%)	56 (60%)	198 (54%)
Race								
White	30 (57%)^γ	36 (69%)	8 (73%)	28 (70%)	21 (66%)	20 (71%)	65 (70%)	245 (67%)
Black or African American	17 (32%)^γ	11 (21%)	3 (27%)	5 (13%)	7 (22%)	7 (25%)	17 (18%)	77 (21%)
Multiracial	2 (4%)^γ	0 (0%)	0 (0%)	1 (3%)	1 (3%)	1 (4%)	1 (1%)	3 (1%)
Other	2 (4%)^γ	2 (4%)	0 (0%)	4 (10%)	1 (3%)	0 (0%)	3 (3%)	19 (5%)
Unknown or not reported	2 (4%)^γ	3 (6%)	0 (0%)	2 (5%)	2 (6%)	0 (0%)	7 (8%)	24 (7%)
Ethnicity								
Unknown or Not Reported	2 (4%)	1 (2%)	0 (0%)	1 (3%)	3 (9%)	2 (7%)	3 (3%)	13 (4%)
Hispanic or Latino	13 (25%)	11 (21%)	0 (0%)	4 (10%)	5 (16%)	2 (7%)	13 (14%)	65 (18%)^β
Not Hispanic or Latino	38 (72%)	40 (77%)	11 (100%)	35 (88%)	24 (75%)	24 (86%)	77 (83%)	290 (79%)^β
**ILLNESS SEVERITY**								
PRISM[a]	10 [3, 16]	9 [3, 16]	11 [5, 27]	7 [3, 15]	10 [0, 18]	11 [6, 21]^β	8 [5, 16]	8 [3, 15]
**INFECTION AT ELIGIBILITY[b]**								
Documented Infection	30 (57%)	34 (65%)	6 (55%)	26 (65%)	21 (66%)	13 (46%)	53 (57%)	210 (57%)
Bacterial	25 (47%)	17 (33%)	4 (36%)	11 (28%)	7 (22%)	8 (29%)	27 (29%)	133 (36%)
Fungal	1 (2%)	3 (6%)	0 (0%)	2 (5%)	1 (3%)	1 (4%)	3 (3%)	4 (1%)
Protozoal	0 (0%)	1 (2%)	0 (0%)	0 (0%)	0 (0%)	0 (0%)	0 (0%)	1 (0%)
Viral[c]	11 (21%)	20 (38%)	2 (18%)	16 (40%)	15 (47%)	5 (18%)	30 (32%)	102 (28%)
**DIAGNOSES**								
ICU Admit Diagnosis								
Cardiac arrest	1 (2%)	3 (6%)	0 (0%)	0 (0%)	0 (0%)	0 (0%)	3 (3%)	12 (3%)
Cardiac disease	0 (0%)	1 (2%)	0 (0%)	2 (5%)	2 (6%)	1 (4%)	4 (4%)	17 (5%)
Liver failure	0 (0%)	0 (0%)	0 (0%)	0 (0%)	0 (0%)	0 (0%)	0 (0%)	5 (1%)

© 2024 Cabler SS et al. *JAMA Network Open*.

eTable 1. Patient Characteristics at ICU Admission by Virus Detection

	EBV+ (N = 53)	CMV+ (N = 52)	HSV-1+ (N = 11)	Adeno+ (N = 40)	BKPyV+ (N = 32)	Parvo+ (N = 28)	HHV-6+ (N = 93)	TTV+ (N = 368)
Renal failure	1 (2%)	2 (4%)	2 (18%)	2 (5%)	1 (3%)	1 (4%)	4 (4%)	8 (2%)
Respiratory failure	19 (36%)	20 (38%)	2 (18%)	18 (45%)	10 (31%)	9 (32%)	37 (40%)	150 (41%)
Seizure	2 (4%)	2 (4%)	0 (0%)	2 (5%)	2 (6%)	2 (7%)	3 (3%)	11 (3%)
Sepsis	24 (45%)	19 (37%)	4 (36%)	11 (28%)	13 (41%)	8 (29%)	29 (31%)	114 (31%)
Other neurologic disease	2 (4%)	0 (0%)	1 (9%)	1 (3%)	0 (0%)	1 (4%)	4 (4%)	14 (4%)
Other	4 (8%)	5 (10%)	2 (18%)	4 (10%)	4 (13%)	6 (21%)	9 (10%)	37 (10%)
**PAST MEDICAL HISTORY**								
Previously healthy	19 (36%)	16 (31%)	6 (55%)	12 (30%)	10 (31%)	13 (46%)	36 (39%)	143 (39%)
Immunocompromised	16 (30%)	20 (38%)	3 (27%)	18 (45%) [γ]	15 (47%) [β]	9 (32%)	35 (38%) [β]	103 (28%)
Malignancy- Hematologic	4 (8%)	8 (15%)	0 (0%)	4 (10%)	8 (25%) [γ]	2 (7%)	12 (13%)	33 (9%)
Malignancy- Solid tumor	4 (8%)	2 (4%)	0 (0%)	1 (3%)	1 (3%)	3 (11%)	6 (6%)	15 (4%)
Hematopoietic cell transplant	4 (8%)	6 (12%)	0 (0%)	8 (20%) [γ]	10 (31%) [γ]	4 (14%)	12 (13%) [γ]	23 (6%)
Solid organ transplant	1 (2%)	2 (4%)	1 (9%)	3 (8%)	0 (0%)	0 (0%)	3 (3%)	10 (3%)
Genetic Disorder/Chromosomal abnormality	11 (21%)	10 (19%)	1 (9%)	6 (15%)	4 (13%)	3 (11%)	16 (17%)	56 (15%)
Metabolic/Mitochondrial disease	2 (4%)	2 (4%)	0 (0%)	4 (10%)	0 (0%)	0 (0%)	3 (3%)	18 (5%)

The table includes counts and summaries for positive viral infections. Viral testing could not be completed on all patients. Patients with failed or missing viral testing were: EBV n=16 patients, CMV n=10, HSV-1 n=16, Adenovirus n=20, BKPyV n=16, Parvo-B19 n=20, HHV-6 n=10, TTV n=10. Comparisons among viral positive vs. negative patients were performed using the Wilcoxon rank sum test or Fisher's exact test as appropriate. Footnotes γ indicate p-values <0.01, β indicate <0.025, and α indicate <0.05.

[a] The worst physiologic values 2 hours prior to ICU admission through 4 hours post ICU admission were used to calculate PRISM.
[b] Patients could have had multiple types of documented infection at eligibility; counts are not mutually exclusive.
[c] Viral infection(s) at eligibility was determined by respective research coordinator chart review and does not include results of investigative viral testing subsequently performed for this study.

© 2024 Cabler SS et al. *JAMA Network Open*.

eTable 2. Patient Characteristics at ICU Admission by Results of Viral Serologic Testing

	EBV Serology			CMV Serology			HSV Serology			HHV-6 Serology		
	Negative (N = 99)	Positive (N = 152)	P-value	Negative (N = 145)	Positive (N = 107)	P-value	Negative (N = 162)	Positive (N = 82)	P-value	Negative (N = 4)	Positive (N = 253)	P-value
**DEMOGRAPHICS**												
Age (years)	6 [3, 12]	10 [6, 14]	0.003[d]	8 [4, 13]	9 [5, 13]	0.270[d]	7 [4, 13]	12 [6, 14]	0.002[d]	6 [4, 9]	9 [5, 13]	0.308[d]
Sex: Male	63 (64%)	77 (51%)	0.051[e]	83 (57%)	57 (53%)	0.608[e]	93 (57%)	44 (54%)	0.588[e]	3 (75%)	140 (55%)	0.632[e]
Race			0.152[e]			0.727[e]			0.985[e]			0.196[e]
White	75 (76%)	100 (66%)		106 (73%)	70 (65%)		115 (71%)	55 (67%)		1 (25%)	177 (70%)	
Black or African American	14 (14%)	34 (22%)		28 (19%)	21 (20%)		31 (19%)	16 (20%)		2 (50%)	48 (19%)	
Multiracial	0 (0%)	2 (1%)		1 (1%)	1 (1%)		2 (1%)	0 (0%)		0 (0%)	2 (1%)	
Other	8 (8%)	7 (5%)		7 (5%)	8 (7%)		10 (6%)	4 (5%)		0 (0%)	15 (6%)	
Unknown or not reported	2 (2%)	9 (6%)		3 (2%)	7 (7%)		4 (2%)	7 (9%)		1 (25%)	11 (4%)	
Ethnicity			0.327[e]			0.332[e]			0.613[e]			1.000[e]
Unknown or Not Reported	3 (3%)	5 (3%)		7 (5%)	1 (1%)		7 (4%)	1 (1%)		0 (0%)	8 (3%)	
Hispanic or Latino	16 (16%)	33 (22%)		24 (17%)	24 (22%)		30 (19%)	18 (22%)		0 (0%)	49 (19%)	
Not Hispanic or Latino	80 (81%)	114 (75%)		114 (79%)	82 (77%)		125 (77%)	63 (77%)		4 (100%)	196 (77%)	
**ILLNESS SEVERITY**												
PRISM[a]	9 [5, 13]	10 [3, 16]	0.486[d]	8 [3, 13]	11 [5, 16]	0.027[d]	9 [5, 15]	10 [3, 15]	0.898[d]	14 [7, 16]	9 [4, 15]	0.566[d]
**INFECTION AT ELIGIBILITY[b]**												
Documented Infection	56 (57%)	79 (52%)	0.518[e]	75 (52%)	61 (57%)	0.444[e]	85 (52%)	46 (56%)	0.684[e]	2 (50%)	137 (54%)	1.000[e]
Bacterial	29 (29%)	60 (39%)	0.472[e]	47 (32%)	43 (40%)	0.407[e]	53 (33%)	32 (39%)	0.457[e]	1 (25%)	92 (36%)	0.639[e]
Fungal	0 (0%)	3 (2%)	1.000[e]	0 (0%)	3 (3%)	0.528[e]	0 (0%)	3 (4%)	0.528[e]	0 (0%)	3 (1%)	--
Protozoal	0 (0%)	0 (0%)	--	0 (0%)	0 (0%)	--	0 (0%)	0 (0%)	--	0 (0%)	0 (0%)	--
Viral[c]	31 (31%)	27 (18%)	0.705[e]	36 (25%)	22 (21%)	1.000[e]	40 (25%)	18 (22%)	0.286[e]	1 (25%)	57 (23%)	1.000[e]
**DIAGNOSES**												
ICU Admit Diagnosis			0.042[e]			0.565[e]			0.003[e]			1.000[e]
Cardiac arrest	3 (3%)	5 (3%)		5 (3%)	2 (2%)		7 (4%)	1 (1%)		0 (0%)	8 (3%)	
Cardiac disease	1 (1%)	5 (3%)		3 (2%)	3 (3%)		3 (2%)	3 (4%)		0 (0%)	6 (2%)	
Liver failure	0 (0%)	1 (1%)		0 (0%)	1 (1%)		0 (0%)	1 (1%)		0 (0%)	1 (0%)	
Renal failure	1 (1%)	6 (4%)		2 (1%)	4 (4%)		1 (1%)	5 (6%)		0 (0%)	7 (3%)	
Respiratory failure	53 (54%)	46 (30%)		63 (43%)	36 (34%)		72 (44%)	24 (29%)		2 (50%)	98 (39%)	

© 2024 Cabler SS et al. *JAMA Network Open*.

eTable 2. Patient Characteristics at ICU Admission by Results of Viral Serologic Testing

	EBV Serology			CMV Serology			HSV Serology			HHV-6 Serology		
	Negative (N = 99)	Positive (N = 152)	P-value	Negative (N = 145)	Positive (N = 107)	P-value	Negative (N = 162)	Positive (N = 82)	P-value	Negative (N = 4)	Positive (N = 253)	P-value
Seizure	2 (2%)	4 (3%)		5 (3%)	2 (2%)		7 (4%)	0 (0%)		0 (0%)	7 (3%)	
Sepsis	27 (27%)	59 (39%)		45 (31%)	42 (39%)		48 (30%)	36 (44%)		5 (50%)	87 (34%)	
Other neurologic disease	3 (3%)	7 (5%)		6 (4%)	4 (4%)		6 (4%)	4 (5%)		0 (0%)	10 (4%)	
Other	9 (9%)	19 (13%)		16 (11%)	13 (12%)		18 (11%)	8 (10%)		0 (0%)	29 (11%)	
**PAST MEDICAL HISTORY**												
Previously healthy	41 (41%)	48 (32%)	0.137[e]	60 (41%)	28 (26%)	0.016[e]	66 (41%)	22 (27%)	0.035[e]	1 (25%)	89 (35%)	1.000[e]
Immunocompromised	14 (14%)	61 (40%)	<.001[e]	29 (20%)	47 (44%)	<.001[e]	35 (22%)	34 (41%)	0.002[e]	1 (25%)	76 (30%)	1.000[e]
Malignancy- Hematologic	3 (3%)	22 (14%)	0.002[e]	7 (5%)	18 (17%)	0.002[e]	7 (4%)	16 (20%)	<.001[e]	0 (0%)	26 (10%)	1.000[e]
Malignancy- Solid tumor	1 (1%)	11 (7%)	0.031[e]	4 (3%)	7 (7%)	0.212[e]	6 (4%)	5 (6%)	0.515[e]	0 (0%)	12 (5%)	1.000[e]
Hematopoietic cell transplant	0 (0%)	17 (11%)	<.001[e]	2 (1%)	15 (14%)	<.001[e]	2 (1%)	14 (17%)	<.001[e]	0 (0%)	17 (7%)	1.000[e]
Solid organ transplant	2 (2%)	6 (4%)	0.485[e]	4 (3%)	4 (4%)	0.726[e]	4 (2%)	4 (5%)	0.448[e]	0 (0%)	8 (3%)	1.000[e]
Genetic Disorder/Chromosomal abnormality	13 (13%)	22 (14%)	0.853[e]	21 (14%)	13 (12%)	0.710[e]	21 (13%)	13 (16%)	0.560[e]	0 (0%)	35 (14%)	1.000[e]
Metabolic/Mitochondrial disease	6 (6%)	6 (4%)	0.548[e]	9 (6%)	4 (4%)	0.566[e]	11 (7%)	1 (1%)	0.065[e]	2 (50%)	11 (4%)	0.013[e]

There were n=108 patients 18 months old or younger excluded from this table. For each serology test, serology-positive patients who received IVIG administration on the same day or before serology sample collection are excluded.

[a] The worst physiologic values 2 hours prior to ICU admission through 4 hours post ICU admission were used to calculate PRISM.
[b] Patients could have had multiple types of documented infection at eligibility; counts are not mutually exclusive.

[c] Viral infection(s) at eligibility was determined by respective research coordinator chart review and does not include results of investigative viral testing subsequently performed for this study.
[d] Wilcoxon rank-sum test.
[e] Fisher's exact test (Monte Carlo approximation for tables larger than 2X2).

© 2024 Cabler SS et al. *JAMA Network Open*.

eTable 3. Comparisons of Herpesvirus PCR and Serology Status

EBV		CMV		HSV		HHV-6	
Viral Status	No. of Patients (row %)	Viral Status	No. of Patients (row %)	Viral Status	No. of Patients (row %)	Viral Status	No. of Patients (row %)
IgG+ / PCR+	39/251 (16%)	IgG+ / PCR+	19/254 (7%)	IgG+ / PCR+	8/246 (3%)	IgG+ / PCR+	61/257 (24%)
IgG + / PCR-	113/251 (45%)	IgG+ / PCR-	88/254 (35%)	IgG+ / PCR-	74/246 (30%)	IgG+ / PCR-	192/257 (75%)
IgG- / PCR+	4/251 (2%)	IgG- / PCR+	11/254 (4%)	IgG- / PCR+	0/246 (0%)	IgG- / PCR+	0/257 (0%)
IgG- / PCR-	95/251 (38%)	IgG- / PCR-	136/254 (54%)	IgG- / PCR-	164/246 (67%)	IgG- / PCR-	4/257 (2%)
IgG+ / PCR+	39/152 (26%)	IgG+ / PCR+	19/107 (18%)	IgG+ / PCR+	8/82 (10%)	IgG+ / PCR+	61/253 (24%)
IgG+ / PCR-	113/152 (74%)	IgG+ / PCR-	88/107 (82%)	IgG+ / PCR-	74/82 (90%)	IgG+ / PCR-	192/253 (76%)
IgG+ / PCR+	39/43 (91%)	IgG+ / PCR+	19/30 (63%)	IgG+ / PCR+	8/8(100%)	IgG+ / PCR+	61/61 (100%)
IgG- / PCR+	4/43 (9%)	IgG- / PCR+	11/30 (37%)	IgG- / PCR+	0/8 (0%)	IgG- / PCR+	0/61 (0%)
IgG+ / PCR+	39/134 (29%)	IgG+ / PCR+	19/155 (12%)	IgG+ / PCR+	8/172 (5%)	IgG+ / PCR+	61/65 (94%)
IgG- / PCR-	95/134 (71%)	IgG- / PCR-	136/155 (88%)	IgG- / PCR-	164/172 (95%)	IgG- / PCR-	4/65 (6%)
IgG+ / PCR-	113/117 (97%)	IgG+ / PCR-	88/99 (89%)	IgG+ / PCR-	74/74 (100%)	IgG+ / PCR-	192/192 (100%)
IgG- / PCR+	4/117 (3%)	IgG- / PCR+	11/99 (11%)	IgG- / PCR+	0/74 (0%)	IgG- / PCR+	0/192 (0%)
IgG+ / PCR-	113/208 (54%)	IgG+ / PCR-	88/224 (39%)	IgG+ / PCR-	74/238 (31%)	IgG+ / PCR-	192/196 (98%)
IgG- / PCR-	95/208 (46%)	IgG- / PCR-	136/224 (61%)	IgG- / PCR-	164/238 (69%)	IgG- / PCR-	4/196 (2%)
IgG- / PCR+	4/99 (4%)	IgG- / PCR+	11/147 (7%)	IgG- / PCR+	0/164 (0%)	IgG- / PCR+	0/4 (0%)
IgG- / PCR-	95/99 (96%)	IgG- / PCR-	136/147 (93%)	IgG- / PCR-	164/164 (100%)	IgG- / PCR-	4/4 (100%)

IgG refers to serology testing. There were n=108 patients 18 months old or younger and excluded. For each serology test, serology-positive patients who received IVIG administration on the same day or before serology sample collection are also excluded. Testing was unable to be performed in 13 patients in EBV, 12 patients with CMV, 20 patients with HSV and 7 patients with HHV-6 due to inadequate sample volume or testing error.

© 2024 Cabler SS et al. *JAMA Network Open*.

eTable 4. Univariable Analysis of Admission Demographic and Clinical Factors Associated With Mortality

	Survived (N = 357)	Died (N = 44)	Odds ratio (95% CI)	P-value
**UNIVARIABLE ANALYSIS**				
Age (years)	5.4 [1.4, 12.0]	7.9 [1.2, 14.5]	1.05 (0.99, 1.11)	0.080
Sex				0.238
Male	194 (87%)	28 (13%)	1.47 (0.78, 2.87)	
Female	163 (91%)	16 (9%)	Reference	
Race				0.377
White	243 (90%)	27 (10%)	Reference	
Other/Not reported	114 (87%)	17 (13%)	1.34 (0.69, 2.54)	
Ethnicity				0.265
Not Hispanic or Latino	282 (88%)	39 (12%)	Reference	
Hispanic or Latino	61 (92%)	5 (8%)	0.59 (0.20, 1.44)	
Unknown or Not Reported	14 (100%)	0 (0%)	--	
PRISM[b]	7.0 [3.0, 13.0]	13.0 [8.5, 18.5]	1.06 (1.02, 1.10)	0.001
Previously healthy	148 (96%)	6 (4%)	0.22 (0.08, 0.50)	<.001
Immunocompromised	87 (81%)	21 (19%)	2.83 (1.49, 5.38)	0.002

[a] Row-wise percentages are presented in this table.
[b] The worst physiologic values 2 hours prior to ICU admission through 4 hours post ICU admission were used to calculate PRISM.

© 2024 Cabler SS et al. *JAMA Network Open*.

eTable 5. Comparisons of EBV PCR and Serology Status and Mortality

EBV			
	**Survived**	**Died**	**P-value**
			0.004[a]
IgG+ / PCR+	33/39 (85%)	6/39 (15%)	
IgG+ / PCR-	96/113 (85%)	17/113 (15%)	
IgG- / PCR+	4/4 (100%)	0/4 (0%)	
IgG- / PCR-	93/95 (98%)	2/95 (2%)	
Incomplete	13/13 (100%)	0/13 (0%)	
			1.000[a]
IgG+ / PCR+	33/39 (85%)	6/39 (15%)	
IgG+ / PCR-	96/113 (85%)	17/113 (15%)	
			1.000[a]
IgG+ / PCR+	33/39 (85%)	6/39 (15%)	
IgG- / PCR+	4/4 (100%)	0/4 (0%)	
			0.008[a]
IgG+ / PCR+	33/39 (85%)	6/39 (15%)	
IgG- / PCR-	93/95 (98%)	2/95 (2%)	
			1.000[a]
IgG+ / PCR-	96/113 (85%)	17/113 (15%)	
IgG- / PCR+	4/4 (100%)	0/4 (0%)	
			0.001[a]
IgG+ / PCR-	96/113 (85%)	17/113 (15%)	
IgG- / PCR-	93/95 (98%)	2/95 (2%)	
			1.000[a]
IgG- / PCR+	4/4 (100%)	0/4 (0%)	
IgG- / PCR-	93/95 (98%)	2/95 (2%)	

IgG refers to serology testing. A postive serology considers EBV, CMV, or HSV tests. There were n=108 patients 18 months old or younger and excluded. There were patients with 'incomplete' serology testing, but these do not contribute to the p-value calculations.
For each serology test, serology-positive patients who received IVIG administration on the same day or before serology sample collection are excluded. Incomplete serology results do not contribute to p-value calculations. Row-wise percentages are presented in this table.
[a] Fisher's exact test

© 2024 Cabler SS et al. *JAMA Network Open*.

eTable 6. Comparisons of CMV PCR and Serology Status and Mortality

CMV			
	**Survived**	**Died**	**P-value**
			0.002[a]
IgG+ / PCR+	13/19 (68%)	6/19 (32%)	
IgG+ / PCR-	77/88 (88%)	11/88 (13%)	
IgG- / PCR+	10/11 (91%)	1/11 (9%)	
IgG- / PCR-	130/136 (96%)	6/136 (4%)	
Incomplete	11/12 (92%)	1/12 (8%)	
			0.076[a]
IgG+ / PCR+	13/19 (68%)	6/19 (32%)	
IgG+ / PCR-	77/88 (88%)	11/88 (13%)	
			0.215[a]
IgG+ / PCR+	13/19 (68%)	6/19 (32%)	
IgG- / PCR+	10/11 (91%)	1/11 (9%)	
			<.001[a]
IgG+ / PCR+	13/19 (68%)	6/19 (32%)	
IgG- / PCR-	130/136 (96%)	6/136 (4%)	
			1.000[a]
IgG+ / PCR-	77/88 (88%)	11/88 (13%)	
IgG- / PCR+	10/11 (91%)	1/11 (9%)	
			0.037[a]
IgG+ / PCR-	77/88 (88%)	11/88 (13%)	
IgG- / PCR-	130/136 (96%)	6/136 (4%)	
			0.427[a]
IgG- / PCR+	10/11 (91%)	1/11 (9%)	
IgG- / PCR-	130/136 (96%)	6/136 (4%)	

IgG refers to serology testing. A postive serology considers EBV, CMV, or HSV tests. There were n=108 patients 18 months old or younger and excluded. There were patients with 'incomplete' serology testing, but these do not contribute to the p-value calculations.
For each serology test, serology-positive patients who received IVIG administration on the same day or before serology sample collection are excluded. Incomplete serology results do not contribute to p-value calculations. Row-wise percentages are presented in this table.
[a] Fisher's exact test

© 2024 Cabler SS et al. *JAMA Network Open*.

eTable 7. Comparisons of HSV-1 PCR and Serology Status and Mortality

HSV-1			
	**Survived**	**Died**	**P-value**
			0.002[a]
IgG+ / PCR+	8/8 (100%)	0/8 (0%)	
IgG+ / PCR-	59/74 (80%)	15/74 (20%)	
IgG- / PCR+	0/0 (0%)	0/0 (0%)	
IgG- / PCR-	155/164 (95%)	9/164 (5%)	
Incomplete	19/20 (95%)	1/20 (5%)	
			0.340[a]
IgG+ / PCR+	8/8 (100%)	0/8 (0%)	
IgG+ / PCR-	59/74 (80%)	15/74 (20%)	
			--
IgG+ / PCR+	8/8 (100%)	0/8 (0%)	
IgG- / PCR+	0/0 (0%)	0/0 (0%)	
			1.000[a]
IgG+ / PCR+	8/8 (100%)	0/8 (0%)	
IgG- / PCR-	155/164 (95%)	9/164 (5%)	
			--
IgG+ / PCR-	59/74 (80%)	15/74 (20%)	
IgG- / PCR+	0/0 (0%)	0/0 (0%)	
			<.001[a]
IgG+ / PCR-	59/74 (80%)	15/74 (20%)	
IgG- / PCR-	155/164 (95%)	9/164 (5%)	
			--
IgG- / PCR+	0/0 (0%)	0/0 (0%)	
IgG- / PCR-	155/164 (95%)	9/164 (5%)	

IgG refers to serology testing. A postive serology considers EBV, CMV, or HSV tests. There were n=108 patients 18 months old or younger and excluded. There were patients with 'incomplete' serology testing, but these do not contribute to the p-value calculations.
For each serology test, serology-positive patients who received IVIG administration on the same day or before serology sample collection are excluded. Incomplete serology results do not contribute to p-value calculations. Row-wise percentages are presented in this table.
[a] Fisher's exact test

© 2024 Cabler SS et al. *JAMA Network Open*.

eTable 8. Comparisons of HHV-6 PCR and Serology Status and Mortality

HHV-6			
	**Survived**	**Died**	**P-value**
			0.060[a]
IgG+ / PCR+	50/61 (82%)	11/61 (18%)	
IgG+ / PCR-	178/192 (93%)	14/192 (7%)	
IgG- / PCR+	0/0 (0%)	0/0 (0%)	
IgG- / PCR-	4/4 (100%)	0/4 (0%)	
Incomplete	7/7 (100%)	0/7 (0%)	
			0.024[a]
IgG+ / PCR+	50/61 (82%)	11/61 (18%)	
IgG+ / PCR-	178/192 (93%)	14/192 (7%)	
			--
IgG+ / PCR+	50/61 (82%)	11/61 (18%)	
IgG- / PCR+	0/0 (0%)	0/0 (0%)	
			1.000[a]
IgG+ / PCR+	50/61 (82%)	11/61 (18%)	
IgG- / PCR-	4/4 (100%)	0/4 (0%)	
			--
IgG+ / PCR-	178/192 (93%)	14/192 (7%)	
IgG- / PCR+	0/0 (0%)	0/0 (0%)	
			1.000[a]
IgG+ / PCR-	178/192 (93%)	14/192 (7%)	
IgG- / PCR-	4/4 (100%)	0/4 (0%)	
			--
IgG- / PCR+	0/0 (0%)	0/0 (0%)	
IgG- / PCR-	4/4 (100%)	0/4 (0%)	

IgG refers to serology testing. A postive serology considers EBV, CMV, or HSV tests. There were n=108 patients 18 months old or younger and excluded. There were patients with ‘incomplete’ serology testing, but these do not contribute to the p-value calculations.
For each serology test, serology-positive patients who received IVIG administration on the same day or before serology sample collection are excluded. Incomplete serology results do not contribute to p-value calculations. Row-wise percentages are presented in this table.
[a] Fisher's exact test

© 2024 Cabler SS et al. *JAMA Network Open*.

eTable 9. Comparison of Ferritin, CRP, and PRISM Across EBV Virus Positivity Cohorts in Vital Status Categories

	EBV					
	S+ / PCR+ (N = 33)	S+ / PCR- (N = 96)	S- / PCR+ (N = 4)	S- / PCR- (N = 93)	Missing (N = 13)	P-value
**PICU Mortality - Alive**						
Max ferritin from blood samples						<.001[1]
n=N	n=33	n=95	n=4	n=93	n=13	
Median [Q1, Q3]	481.0 [172.6, 718.0]	321.0 [134.0, 1011.9]	114.0 [48.5, 304.0]	192.8 [92.0, 302.0]	183.0 [77.0, 624.0]	
Max CRP						0.047[1]
n=N	n=33	n=96	n=4	n=93	n=13	
Median [Q1, Q3]	19.9 [12.4, 36.8]	18.7 [8.9, 27.7]	9.2 [4.0, 19.9]	13.9 [5.4, 25.4]	9.0 [6.4, 27.6]	
Total PRISM score						0.126[1]
n=N	n=33	n=96	n=4	n=93	n=13	
Median [Q1, Q3]	11.0 [6.0, 16.0]	8.0 [3.0, 14.0]	2.5 [1.0, 7.0]	9.0 [5.0, 13.0]	11.0 [0.0, 16.0]	
**PICU Mortality - Dead**						
Max ferritin from blood samples						0.030[1]
n=N	n=6	n=17	n=0	n=2		
Median [Q1, Q3]	1070.5 [509.0, 1705.0]	3044.0 [1104.0, 4454.0]	- [-, -]	83.0 [68.0, 98.0]		
Max CRP						0.454[1]
n=N	n=6	n=17	n=0	n=2		
Median [Q1, Q3]	23.9 [9.6, 37.4]	31.6 [17.0, 39.4]	- [-, -]	14.1 [7.5, 20.8]		
Total PRISM score						0.908[1]
n=N	n=6	n=17	n=0	n=2		
Median [Q1, Q3]	14.5 [0.0, 20.0]	14.0 [9.0, 19.0]	- [-, -]	13.5 [10.0, 17.0]		

[1] Kruskal-Wallis test.

© 2024 Cabler SS et al. *JAMA Network Open*.

eTable 10. Comparison of Ferritin, CRP, and PRISM Across CMV Virus Positivity Cohorts in Vital Status Categories

	CMV					
	S+ / PCR+ (N = 13)	S+ / PCR- (N = 77)	S- / PCR+ (N = 10)	S- / PCR- (N = 128)	Missing (N = 11)	P-value
**PICU Mortality - Alive**						
Max ferritin from blood samples						<.001[1]
n=N	n=12	n=77	n=10	n=128	n=11	
Median [Q1, Q3]	690.8 [172.0, 2745.0]	370.0 [183.0, 1007.0]	278.0 [92.0, 443.0]	184.0 [94.5, 327.1]	569.0 [77.0, 783.0]	
Max CRP						0.017[1]
n=N	n=13	n=77	n=10	n=128	n=11	
Median [Q1, Q3]	25.1 [9.1, 36.8]	22.1 [11.0, 27.9]	14.9 [3.7, 25.6]	13.5 [5.4, 22.6]	32.0 [7.4, 49.0]	
Total PRISM score						0.291[1]
n=N	n=13	n=77	n=10	n=128	n=11	
Median [Q1, Q3]	6.0 [2.0, 15.0]	11.0 [5.0, 15.0]	9.5 [3.0, 15.0]	7.5 [3.0, 13.0]	11.0 [5.0, 21.0]	
**PICU Mortality - Dead**						
Max ferritin from blood samples						0.147[1]
n=N	n=6	n=11	n=1	n=6	n=1	
Median [Q1, Q3]	2118.8 [1161.0, 4454.0]	2036.1 [141.0, 6361.0]	117.0 [117.0, 117.0]	755.0 [88.3, 1705.0]	4380.0 [4380.0, 4380.0]	
Max CRP						0.448[1]
n=N	n=6	n=11	n=1	n=6	n=1	
Median [Q1, Q3]	31.7 [24.2, 45.0]	19.9 [6.3, 39.4]	9.6 [9.6, 9.6]	21.1 [20.8, 26.8]	41.4 [41.4, 41.4]	
Total PRISM score						0.454[1]
n=N	n=6	n=11	n=1	n=6	n=1	
Median [Q1, Q3]	16.0 [3.0, 20.0]	13.0 [10.0, 23.0]	19.0 [19.0, 19.0]	8.0 [0.0, 17.0]	18.0 [18.0, 18.0]	

[1] Kruskal-Wallis test.

© 2024 Cabler SS et al. *JAMA Network Open*.

eTable 11. Comparison of Ferritin, CRP, and PRISM Across HSV-1 Virus Positivity Cohorts in Vital Status Categories

	HSV					
	S+ / PCR+ (N = 8)	S+ / PCR- (N = 59)	S- / PCR+ (N = 0)	S- / PCR- (N = 153)	Missing (N = 19)	P-value
**PICU Mortality - Alive**						
Max ferritin from blood samples						<.001[1]
n=N	n=8	n=58	n=0	n=153	n=19	
Median [Q1, Q3]	354.5 [101.5, 495.0]	490.8 [212.0, 1397.7]	- [-, -]	187.0 [94.0, 329.0]	487.0 [77.0, 806.0]	
Max CRP						<.001[1]
n=N	n=8	n=59	n=0	n=153	n=19	
Median [Q1, Q3]	12.8 [8.0, 19.8]	25.6 [12.4, 32.6]	- [-, -]	13.5 [5.4, 22.0]	23.2 [6.4, 35.1]	
Total PRISM score						0.343[1]
n=N	n=8	n=59	n=0	n=153	n=19	
Median [Q1, Q3]	10.0 [5.0, 14.0]	6.0 [2.0, 14.0]	- [-, -]	9.0 [5.0, 15.0]	6.0 [0.0, 15.0]	
**PICU Mortality - Dead**						
Max ferritin from blood samples						0.069[1]
n=N	n=0	n=15	n=0	n=9	n=1	
Median [Q1, Q3]	- [-, -]	3044.0 [1104.0, 6361.0]	- [-, -]	1001.0 [117.0, 1829.0]	98.0 [98.0, 98.0]	
Max CRP						0.655[1]
n=N	n=0	n=15	n=0	n=9	n=1	
Median [Q1, Q3]	- [-, -]	31.6 [12.7, 45.0]	- [-, -]	21.1 [20.8, 37.4]	7.5 [7.5, 7.5]	
Total PRISM score						0.199[1]
n=N	n=0	n=15	n=0	n=9	n=1	
Median [Q1, Q3]	- [-, -]	15.0 [10.0, 23.0]	- [-, -]	10.0 [6.0, 18.0]	10.0 [10.0, 10.0]	

[1] Kruskal-Wallis test.

© 2024 Cabler SS et al. *JAMA Network Open*.

eTable 12. Comparison of Ferritin, CRP, and PRISM Across HHV-6 Virus Positivity Cohorts in Vital Status Categories

	HHV6					
	S+ / PCR+ (N = 50)	S+ / PCR- (N = 178)	S- / PCR+ (N = 0)	S- / PCR- (N = 4)	Missing (N = 7)	P-value
**PICU Mortality - Alive**						
Max ferritin from blood samples						0.423[1]
n=N	n=49	n=178	n=0	n=4	n=7	
Median [Q1, Q3]	226.0 [123.0, 1358.0]	228.6 [113.0, 487.0]	- [-, -]	231.5 [93.5, 704.6]	147.0 [69.0, 783.0]	
Max CRP						0.158[1]
n=N	n=50	n=178	n=0	n=4	n=7	
Median [Q1, Q3]	21.3 [9.9, 28.8]	14.4 [6.8, 26.2]	- [-, -]	24.5 [11.8, 36.8]	22.8 [6.4, 52.3]	
Total PRISM score						0.555[1]
n=N	n=50	n=178	n=0	n=4	n=7	
Median [Q1, Q3]	8.5 [5.0, 16.0]	8.0 [3.0, 13.0]	- [-, -]	13.5 [6.5, 15.5]	15.0 [0.0, 21.0]	
**PICU Mortality - Dead**						
Max ferritin from blood samples						0.055[1]
n=N	n=11	n=14	n=0	n=0		
Median [Q1, Q3]	1829.0 [1140.0, 14055.0]	1052.5 [100.0, 3044.0]	- [-, -]	- [-, -]		
Max CRP						0.067[1]
n=N	n=11	n=14	n=0	n=0		
Median [Q1, Q3]	37.4 [17.0, 45.0]	20.9 [7.5, 31.7]	- [-, -]	- [-, -]		
Total PRISM score						0.742[1]
n=N	n=11	n=14	n=0	n=0		
Median [Q1, Q3]	18.0 [0.0, 20.0]	13.0 [10.0, 19.0]	- [-, -]	- [-, -]		

[1] Kruskal-Wallis test.

© 2024 Cabler SS et al. *JAMA Network Open*.
