# Supplementary material for: Viral DNAemia and DNA Virus Seropositivity and Mortality in Pediatric Sepsis
Source: JAMA Netw Open. 2024 Feb 26;7(2):e240383. doi: 10.1001/jamanetworkopen.2024.0383 (PMC10897747; doi:10.1001/jamanetworkopen.2024.0383)
Supplement: Supplement 2. — Data Sharing Statement [file jamanetwopen-e240383-s002.pdf]

Data Sharing Statement

Cabler. Viral DNAemia and DNA Virus Seropositivity and Mortality in Pediatric Sepsis. *JAMA Netw Open*. Published February 26, 2024. doi:10.1001/jamanetworkopen.2024.0383

Data
Data available: Yes
Data types: Deidentified participant data, Data dictionary
How to access data: The data is available from the DASH website maintained by the NICHD Permission must be requested for IRB approval from the University of Utah Data Coordinating Center for the NICHD Collaborative Pediatric Critical Care Research Network
When available: With publication

Supporting Documents
Document types: None

Additional Information
Who can access the data: Researchers whose proposed use of the data has been approved by the University of Utah Data Coordinating Center for the NICHD Collaborative Pediatric Critical Care Research Network
Types of analyses: Any
Mechanisms of data availability: Without investigator support, after approval of a proposal, with signed data access agreement
